# The colistin resistance pandemic: A dual threat from plasmid-encoded mobilised colistin resistance genes and chromosomal mutations

**DOI:** 10.4102/ajlm.v15i1.3122

**Published:** 2026-06-12

**Authors:** Muzaheed

**Affiliations:** 1 Department of Clinical Laboratory Science, College of Applied Medical Sciences, Imam Abdulrahman Bin Faisal University, Dammam, Saudi Arabia

**Keywords:** colistin resistance, *mcr* genes, plasmid-mediated resistance, chromosomal mutations, One Health

## Abstract

**Background:**

Colistin remains a critical last-line therapeutic option against multidrug-resistant Gram-negative infections; but its utility is threatened increasingly by the global spread resistance determinants. Resistance emerges through two principal genetic pathways: horizontal transmission of *mcr* genes enabling rapid interspecies dissemination, and vertical chromosomal mutations facilitating clonal expansion.

**Aim:**

To compare plasmid-mediated and chromosomal colistin resistance mechanisms and examine their clinical significance, epidemiological implications, diagnostic challenges, and surveillance barriers within a One Health framework.

**Methods:**

For this narrative, non-systematic review, searches were conducted in PubMed, MEDLINE, Web of Science, Scopus and African Journals Online (AJOL) to identify studies on colistin resistance, its emergence, clinical implications, and related surveillance and diagnostic barriers.

**Results:**

Several genetic determinants influence colistin susceptibility; notably, *mcr* variants and chromosomal mutations in Lipid A modification genes (e.g., *pmrA, pmrB* and *mgrB*) reduce colistin binding and bactericidal activity. In African and other resource-limited settings, limited laboratory infrastructure further complicates detection and surveillance, underscoring the need for context-appropriate diagnostics and antimicrobial stewardship. Collectively, these factors pose major clinical and epidemiological concerns.

**Conclusion:**

Although surveillance-driven stewardship may mitigate resistance trends, its effectiveness is constrained by the convergence of colistin and carbapenem resistance, increasing the risk of pan-drug-resistant infections. Rapid field-deployable diagnostics and harmonised global surveillance systems that consider the distinct evolutionary trajectories of plasmid-mediated and chromosomal resistance remain urgently needed.

**What this study adds:**

This review highlights the contrasting evolutionary pathways of plasmid-mediated and chromosomal colistin resistance and emphasises the importance of integrated surveillance, diagnostics, and stewardship strategies within a One Health approach.

## Introduction

The World Health Organization (WHO) recognises antimicrobial resistance (AMR) as one of the top global threats, which exposes millions to the risk of untreatable infections by limiting vital life-saving treatment alternatives.^1^ Based on 2021 data, of the 4.71 million overall deaths, 1.14 million are caused directly by bacterial AMR. This figure is projected to reach nearly 8.22 million annually by 2050. AMR-related deaths are associated with eight major pathogens: *Escherichia coli, Acinetobacter baumannii, Neisseria gonorrhoeae, Klebsiella pneumoniae, Shigella* spp., *non-typhoidal Salmonella* spp., *Staphylococcus aureus*, and *Streptococcus pneumoniae*.^2,3^

Broad-spectrum β-lactams, particularly carbapenems, are used frequently against severe infections attributable to multidrug resistant (MDR) Gram-negative bacteria.^4^ However, the emergence of Gram-negative carbapenem-resistant bacteria has limited the effectiveness of these broad-spectrum antibiotics. Despite former restrictions on colistin owing to its adverse nephrotoxic and neurotoxic effects, this older antibiotic (discovered in 1949) has now become a ‘last-resort’ or ‘salvage therapy’ option for treating carbapenem-resistant infections.^5^

**FIGURE 1:**
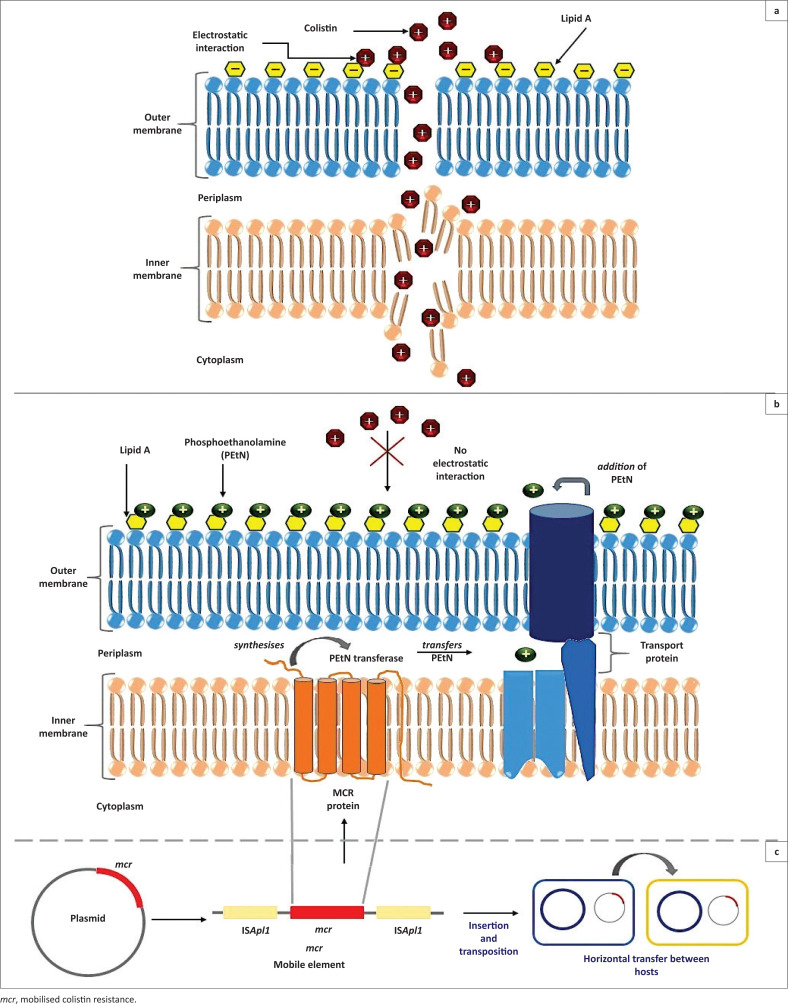
Schematic representation of plasmid-mediated colistin resistance; (a) Colistin binding to the Lipid A component of lipopolysaccharides in susceptible bacteria, (b) *mcr*-mediated modification of Lipid A with phosphoethanolamine, reducing colistin affinity, (c) Possible horizontal gene transfer routes among animals, humans, and environmental reservoirs under antimicrobial selection pressure.

By electrostatic binding to negatively charged Lipid A component of lipopolysaccharide (LPS) in the outer membrane, colistin, a cationic peptide, also known as polymyxin E, exerts its antibacterial effect by disrupting membrane integrity and increasing permeability (Figure 1). Therefore, AMR development in Gram-negative bacteria, which employ diverse resistance mechanisms against both colistin and carbapenem, poses significant therapeutic challenges in clinical settings.^6^ These bacteria acquire colistin resistance via two modes: plasmid-mediated or chromosomal.

Lipopolysaccharide biogenesis-related chromosomal gene mutations, along with subsequent modifications that increase cationic groups (i.e. an extra positive charge on Lipid A), diminish the interaction of colistin with Lipid A. This confers chromosome-mediated colistin resistance. In plasmid-based colistin resistance, the scientific and medical community faces a significant challenge currently, since associated mobile colistin resistance (*mcr*) genes alter Lipid A in most Gram-negative bacteria and rapidly transfer horizontally among different Enterobacterales genera. Plasmid-mediated resistance disseminates rapidly through horizontal transfer between bacterial species, while chromosomal resistance is transmitted stably in clonal lineages.^7^ These mechanisms diminish the potency of existing antimicrobial therapies and increase the clinical complexity of such infections.

Overuse or misuse of antibiotics in human and animal medicine exerts selection pressure on bacteria, accelerating the emergence and spread of multidrug-resistant (MDR) strains. Therefore, this AMR challenge in community and clinical settings has emphasised the importance of the ‘One Health’ approach, where collective efforts are required at local, national and global levels to ensure human, animal and environmental health, alongside stringent strategies to minimise antibiotic overuse and misuse of antibiotics to mitigate the risk of development of antibiotic resistance in bacteria.^7,8^

While colistin resistance has been documented globally, the situation in African countries remains incompletely understood. Studies from Egypt, Nigeria, South Africa, and Cameroon have reported *mcr-1* and related variants in both clinical and agricultural isolates, although large-scale surveillance data remain scarce across much of the continent.^9,10,11,12^

The Africa CDC (Centre for Disease Control) AMR Surveillance Network and the AU-IBAR (African Union Inter-African Bureau for Animal Resources) represent important steps toward coordinated resistance monitoring, but laboratory capacity, funding, and data-sharing infrastructure remain uneven.^13^

This review provides a comprehensive comparison of plasmid-mediated and chromosomal colistin resistance mechanisms, highlights their clinical and epidemiological impacts, discusses associated diagnostic and surveillance challenges, and synthesises key mitigation strategies within a crucial One Health framework.

## Search strategy and selection criteria

The review was conducted as a narrative, non-systematic synthesis. Because the scope is broad, PRISMA methodology was not applied. Four databases were searched: PubMed/MEDLINE, Web of Science, Scopus, and African Journals Online (AJOL), covering January 2015 to March 2025. Inclusion criteria were: peer-reviewed, English-language publications on colistin/polymyxin B resistance mechanisms, epidemiology, diagnostics, or clinical outcomes in global or African settings. No formal quality scoring was performed.

## Plasmid-mediated colistin resistance

### Discovery of the mobilised colistin resistance gene variants

The first identification of a colistin-resistant *Escherichia coli* strain, from the pig sample in China in 2015 revealed that widespread colistin use in livestock farming probably exerts selective pressure on gut bacteria, promoting the emergence and rapid spread of the plasmid-mediated *mcr-1* gene associated with colistin resistance. After the first discovery of the *mcr*-1 gene in China in 2015, extensive studies on *mcr* genes from 2016 to 2020 have detected additional *mcr* gene variants, worldwide. From other *mcr* gene variants, *mcr*-3, -7, -8 and -10 were first detected in China, while *mcr*- 2, -4, -5 and -6 were first reported in European regions and the *mcr*-9 gene was first discovered in the US.^14^ All these gene variants were detected in different microorganisms isolated from different hosts, as depicted in Table 1. Among diverse bacterial species from humans, animals, and the environment, 10 *mcr* gene variants (*mcr-1* to *mcr-10*) have been identified, each conferring distinct colistin resistance mechanisms among members of the *Enterobacteriaceae* family. Although the catalytic activity and substrate specificity differ among phosphoethanolamine transferase encoded by various *mcr* variants, all share a conserved function in Lipid A modification.^15,16^

**TABLE 1:** Historical timeline about the discovery of novel *mcr -1* to *mcr -10* gene variants.

*mcr* gene variant	First detection of *mcr* gene variants				Reference
	Region or country	Year	Bacteria	Sample or host	
*mcr-1*	China	2015	*Escherichia coli*	Pigs and humans	^17^
*mcr-2*	Belgium	2016	*Escherichia coli*	Pigs and calves	^18^
*mcr-3*	China	2017	*Escherichia coli*	Pigs	^19^
*mcr-4*	Italy Spain Belgium	2013 2015 2016	*Salmonella enterica*, and *Escherichia coli*	Pigs	^20^
*mcr-5*	Germany	2017	*Salmonella enterica* subsp. *enterica* serovar Paratyphi B	Poultry and food	^21^
*mcr-6*	Great Britain	2017	Moraxella pluranimalium	Pigs	^22^
*mcr-7*	China	2018	Klebsiella pneumoniae	Chickens	^23^
*mcr-8*	China	2018	Klebsiella pneumoniae	Livestock and humans	^24^
*mcr-9*	US	2019	*Salmonella enterica* serotype Typhimurium	Human	^25^
*mcr-10*	China	2016	Enterobacter roggenkampii	Humans	^26^

Note: Please see the full reference list of Muzaheed. The colistin resistance pandemic: A dual threat from plasmid-encoded mobilised colistin resistance genes and chromosomal mutations. Afr J Lab Med. 2026;15(1), a3122. https://doi.org/10.4102/ajlm.v15i1.3122.

*mcr*, mobilised colistin resistance.

### Biochemical mechanism and dynamics of genetic mobilisation

The phosphoethanolamine transferase (PET) enzyme modifies the Lipid A component of LPS by adding cationic groups, thereby reducing colistin binding and conferring resistance (Figure 1). Among the various *mcr* variants, *mcr-1* is the one most extensively studied and is located on multiple conjugative and non-conjugative plasmids, including IncX4, IncH1, IncI2, IncHI1, and IncHI2, as well as on hybrid plasmids such as IncX3–X4 and IncI2–IncFIB.^11^ Plasmid analyses revealed *ISApl1*, an *IS30* transposase, flanking the *mcr-1*, as part of the composite transposon *Tn6330* (*ISApl1–mcr-1–ISApl1*), which facilitates *mcr-1* mobilisation onto IncHI2 and IncI2 plasmids.^27^ This insertion sequence aids *mcr-1* gene mobilisation and rapid dissemination of colistin resistance among different *Enterobacterales* species,^27^ as illustrated in Figure 1. In the IncX4 plasmid of *K. pneumoniae*, the *mcr-1–pap2* region forms a hairpin structure flanked by two *ISApl1* elements, serving potentially as an intermediate that facilitates *mcr-1* dissemination. A subsequent loss of *ISApl1* in IncX4 plasmid may enhance *mcr-1* gene stability and provide adaptive advantages to host bacteria under selective antibiotic pressure.^28^ In contrast, *mcr-9* and *mcr-10* genes identified in 59 *Enterobacter* isolates were carried on a variety of Inc plasmid types, including single replicons such as IncFIB(pECLA) and IncFIB(K), and multiple replicons such as IncFIA(HI1)–IncFIB(K) and IncFIB(pECLA)–IncFII(pECLA).^29^

### Co-resistance with other antibiotic classes

Owing to the presence of co-resistant genes on plasmids and chromosomes, bacteria have developed resistance to carbapenems, colistin, and β-lactam antibiotics, leading to extensively drug-resistant (XDR), multidrug-resistant (MDR), and pandrug-resistant (PDR) phenotypes in clinical, community and environmental settings.^30^

Genomic sequencing studies reveal that plasmids carrying the *mcr-1* gene often co-harbour other resistance determinants, including carbapenemase and extended-spectrum β-lactamase (ESBL) genes. IncHI2 plasmids harbouring *mcr-1* often carry additional resistance genes against multiple antibiotic classes, including β-lactams (*blaTEM−1*), aminoglycosides (*aadA1, aadA2, aph(6)-Id*), tetracyclines (*tetA, tetR*), macrolides *(mef(B*)), and sulphonamides (*sul1, sul2, sul3*). Other variants, such as *mcr-4* and *mcr-9*, have been associated with co-resistance to carbapenemase genes *KPC-2* and *OXA-48*.^31^ Coexistence of the *mcr-1* gene with other antimicrobial resistant genes (ARGs) such as *floR, tetA, fosA, aac-3-IV, aac(6_)-lb, aadA1*, ^*bla*^*TEM* has been documented in many colistin-resistant *Enterobacteriaceae* like *Salmonella* spp., *E. coli*, and *K. pneumoniae*. Such coexistence heightens the risk of the emergence of pan-drug resistance and potentially threatens the use of active therapeutics for such infections.^32^ Co-occurrence of distinct *mcr* genes, *mcr-1, mcr-3* and *mcr-7*, in addition to different ARGs against various antibiotics comprising β-lactams (*bla_CTX-M-Gp1-9_, bla_VEB_, bla_CMY_ bla_PER_*), tetracyclines (*tetA, tetB, tetC*), aminoglycosides (*aac(6′)-Ib, aadA, aph(3′)-Ia, ant(2′)-Ia*), quinolones (*oqxA, oqxB, qnrA, qnrB, qnrS*), sulphonamides (*sul1, sul2, sul3*), chloramphenicol (*cmlA*), and macrolides (*ermB, mefAE*) were observed in zoo animals. Co-presence of many ARGs points out that a zoo acts as a potential reservoir contributing multidrug resistance and the possibility of its dissemination in other settings.^33^

The convergence of colistin and carbapenem resistance in a single isolate is of particular clinical concern. For hospitals in African settings, where newer β-lactam combinations and carbapenemase inhibitors are often unavailable or unaffordable, pan-drug-resistant infections can leave clinicians with no viable treatment option.^34^ This scenario, already documented in intensive care unit (ICU) outbreaks across several countries, underscores why containing the spread of co-resistant strains requires urgent and coordinated action.^35^

### Global epidemiology and dissemination

A systematic review conducted between 2014 and 2021 on the global prevalence of plasmid-mediated *mcr* genes in *E. coli* across 54 countries and five continents reported an overall prevalence of 6.51%. The Asian region exhibited the greatest diversity of *mcr* variants (*mcr-1* to *mcr-9*, excluding *mcr*-2), followed by Europe, which reported five variants (*mcr-1* to *mcr-5*). The use of colistin as a growth promoter in pig farming may contribute to the diverse distribution of *mcr* variants observed in these regions.^36^ Oceania documented only the *mcr-1* gene variant, while the American region reported two variants: *mcr-1* and *mcr-3*. Africa recorded three *mcr* gene variants: *mcr-1, mcr-5* and *mcr-8*. In Asia, Thailand demonstrated the highest diversity with six variants (*mcr-1, mcr-3*, and *mcr-6* to *mcr-9*), whereas in Europe, Spain exhibited four variants (*mcr-1* and *mcr-3* to *mcr-5*).^36,37,38,39^ Among livestock, *mcr* prevalence was highest in pigs (14.9%) and chickens (15.8%). Conversely, isolates from healthy humans exhibited lower *mcr* prevalence rates of 7.4% and clinical cases of 4.2%.^36^ In the African region, *mcr-1* to *mcr-3, mcr-4, mcr-5, mcr-8* and *mcr-9* variants were reported in different pathogens including *E. coli, Alcaligenes faecalis Klebsiella, Citrobacter, Salmonella, Enterobacter, Pseudomonas*, and *Acinetobacter* species, emphasising the need for strategies to constrain the spread of colistin resistance in Africa.^40^ In Latin America, *mcr-1* prevalence was reported in bacterial isolates from animals (8.7%), food (5.4%), and humans (2.0%).^41^ Environmental studies have detected colistin-resistant bacteria in migratory bird faecal samples, suggesting that birds act as vectors in the environmental circulation of *mcr-1* through contact with contaminated habitats.^42^ Anthropogenic activities such as agricultural runoff and marine pollution may serve further as reservoirs and dissemination pathways for colistin resistance in aquatic ecosystems.^43^ These findings highlight the complex interplay between human, animal, and environmental factors driving the occurrence, contamination, and global dissemination of plasmid-mediated *mcr* genes, raising serious public health concerns.

### African epidemiology of mobilised colistin resistance genes

Data on *mcr* gene distribution in Africa are growing but remain fragmentary relative to other regions. In North Africa, *mcr-1* has been identified in clinical *E. coli* and *K. pneumoniae* isolates from Egypt, Algeria, and Tunisia.^44,45^

In sub-Saharan Africa, studies from Nigeria and South Africa have detected *mcr-1* in human clinical samples and livestock. Additional *mcr* variants including *mcr-5, mcr-8, mcr-9*, and *mcr-10* have been identified in Nigeria, while *mcr-8.1* has been reported from Kenya, frequently alongside extended-spectrum β lactamases (ESBL)-producing strains.^40,46^

The widespread informal use of colistin in African veterinary settings, combined with limited regulatory enforcement, creates conditions for ongoing resistance selection and spread.^47,48^ The absence of Africa from multicentre clinical outcome studies represents a critical knowledge gap that warrants urgent attention from researchers and policymakers on the continent.^49^

## Chromosomal colistin resistance

### Genetic mutations: Interplay of two-component systems

In *Enterobacteriaceae*, chromosomally mediated reduction in colistin (polymyxin) susceptibility is linked primarily to chemical modifications of the Lipid A moiety of lipopolysaccharide in the bacterial outer membrane. The addition of amino-4-deoxy-L-arabinose (L-Ara4N), phosphoethanolamine (PEtN), or both to Lipid A decreases its affinity for colistin. Environmental stimuli activate signal transduction pathways that regulate these chromosomal adaptations. These adaptive responses induce L-Ara4N and/or PEtN modification of Lipid A through overexpression of the *pmrHFIJKLM* (also known as *arnBCADTEF* or *pbgPE*), *pmrCAB*, and *pmrE* operons. The expression of these operons is regulated by two-component systems (TCSs), namely PmrA–PmrB and PhoP–PhoQ. In these systems, the *pmrCAB* operon encodes the sensor kinase PmrB and its cognate response regulator PmrA, which can be activated by the PhoP–PhoQ system.^50^ In the presence of polymyxins, low Mg^2+^ concentrations, or acidic conditions, the PhoP–PhoQ TCS activates Lipid A-modifying genes, thereby reducing colistin binding. Upon activation, the sensor kinase PhoQ undergoes autophosphorylation and subsequently phosphorylates its response regulator PhoP. The phosphorylated PhoP activates PmrD, in turn stimulating the PmrA–PmrB system, facilitating Lipid A modification. Under conditions of elevated Fe^3+^ or Al^3+^ concentrations and low pH, autophosphorylated PmrB transfers the phosphate group to PmrA, initiating transcription of the *arnBCADTEF* (*pbgPE*), *pmrCAB*, and *pmrE* operons. The *pmrE* gene product catalyses the synthesis and transfer of L-Ara4N to Lipid A. Meanwhile, the *pmrC* gene within the *pmrCAB* operon encodes PET, which facilitates the incorporation of positively charged PEtN into Lipid A.^50,51^ Together, the PhoP–PhoQ and PmrA–PmrB systems modify the Lipid A moiety with L-Ara4N and PEtN, thereby reducing its overall negative charge. This reduction in negative charge weakens the electrostatic interactions between colistin and Lipid A, providing protection under antibiotic selection pressure.^52^ In another TCS, CrrA–CrrB, a missense mutation can induce autophosphorylation of the sensor kinase CrrB, which then phosphorylates its cognate regulator CrrA, leading to synthesis of the CrrC protein. The CrrC protein, acting via the PmrA–PmrB TCS, promotes the production of L-Ara4N and PEtN.^53^ Such TCS-regulated chromosomal mutations confer colistin resistance in various bacteria, including *Enterobacter* spp., *K. pneumoniae, Salmonella* spp., *E. coli, P. aeruginosa*, and *A. baumannii*.^54^

### Mutation-based suppression of inhibitor gene

The small transmembrane protein MgrB serves as a negative regulator of the PhoP–PhoQ TCS. Upon environmental stimulation, PhoQ phosphorylates PhoP, which activates the transcription of multiple genes, including *mgrB*. MgrB inhibits PhoQ activity, suppressing PhoP phosphorylation and downregulating the PhoP–PhoQ signalling cascade.^55^ In *K. pneumoniae*, deleterious mutations in the *mgrB* gene disrupt its inhibitory role on the PhoP–PhoQ TCS, thereby promoting colistin resistance. Nonsense mutations, deletions, insertion sequence (IS) transpositions, and amino acid substitutions can lead to a dysfunctional mgrB, resulting in upregulation of PhoP-regulated genes that enhance Lipid A modification and confer colistin resistance.^56,57^

### Mutations in Lipid A biosynthesis genes and fitness cost

Alternatively, deletions or insertions in Lipid A biosynthesis genes (*lpxA, lpxC*, and *lpxD*) disrupt Lipid A and, consequently, LPS production, promoting colistin heteroresistance in *A. baumannii*.^58^ However, LPS loss reduces bacterial survival, virulence, and transmission capacity, imposing a higher fitness cost compared with *PmrA–PmrB*-mediated resistance.^59^ In general, chromosomal mutation-mediated colistin resistance is more stable but carries a lower horizontal transmission risk than plasmid-mediated resistance.^60^ Nevertheless, studies have reported vertical transmission of co-integrated *mcr* genes from plasmids to chromosomes through *ISApl1* transposons, integrative and conjugative elements (ICEs), or insertion sequences. Following integration, these elements can be maintained stably in the chromosomes of daughter cells, as observed in resistant strains of *E. coli* and *Ralstonia pickettii*. In addition, inactivation of the chromosomal *mgrB* gene via *ISKpn72* mobile element insertion generates more stable resistant genotypes in *K. pneumoniae*. Such genetic events pose significant challenges to controlling the dissemination of colistin-resistant infections.^61,62,63,64^ Clonal outbreaks of chromosomally mediated colistin- and carbapenem-resistant infections in ICUs often lead to treatment failure and prolonged hospitalisation, underscoring the urgent need for effective containment strategies.^65,66,67^

**FIGURE 2:**
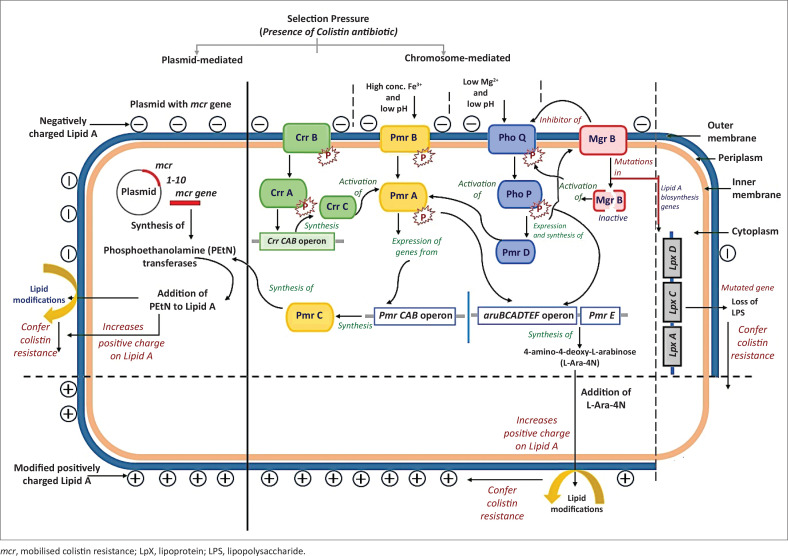
Schematic representation of plasmid- and chromosome-mediated mechanisms of colistin resistance in Gram-negative bacteria. The left panel illustrates *mcr*-encoded phosphoethanolamine (pEtN) transferase activity leading to Lipid A modification, while the right panel depicts chromosomal regulatory systems (PhoP/PhoQ, PmrA/PmrB, CrrA/CrrB) and *mgrB* mutations that promote Lipid A modifications and reduce colistin binding. Loss of lipopolysaccharides (LPS) or Lipid A biosynthesis genes can also confer high-level resistance.

Both plasmid-mediated and chromosomal mutation-based mechanisms contribute to colistin heteroresistance in Gram-negative bacteria (Figure 2), collectively posing a significant healthcare burden.

## Clinical implications

### Therapeutic strategies in the context of colistin resistance

Colistin is commonly used to treat carbapenem-resistant infections, either as monotherapy or in combination with other antibiotics; however, current clinical evidence remains inconclusive regarding which approach is more effective.^68,69^ The emergence of colistin resistance, often alongside resistance to other antibiotic classes, poses a serious threat owing to the growing prevalence of pandrug-resistant (PDR) bacterial strains.^70,71^ To address this therapeutic challenge, several strategies are being explored, including the development of novel drugs, combination therapies involving colistin and other agents, drug repurposing, photodynamic therapy, colistin-sparing regimens, nanotechnology-based delivery systems, phage therapy, CRISPR (Clustered Regularly Interspaced Short Palindromic Repeats) interference (CRISPRi), and quorum-quenching (quorum-sensing inhibition) approaches. Most of these strategies remain in the *in vitro* or preclinical phase, with limited supporting evidence from human clinical trials. Although these innovations show promise, further validation through well-designed clinical studies is essential before they can be implemented as alternative therapies against colistin resistance.^72,73^ A colistin-sparing approach can be adopted to treat carbapenem-resistant *Enterobacteriaceae* (CRE) infections using newer antimicrobial agents such as cefiderocol and sulbactam/durlobactam (novel β-lactam antibiotics), plazomicin (a next-generation aminoglycoside), eravacycline (a fluorocycline antibiotic), and omadacycline (an aminomethylcycline antibiotic). Fosfomycin has also demonstrated potent antimicrobial activity against MDR *Enterobacteriaceae*-related infections.^74,75^ These alternative antibiotics can be administered either as monotherapy or in combination with other agents to reduce dependence on colistin for treating CRE infections and to limit the emergence of further colistin resistance.^76^

Combination therapy involving colistin with carbapenems or β-lactamase inhibitors has demonstrated synergistic effects against MDR and XDR *Acinetobacter baumannii* infections, as well as significant *in vitro* antimicrobial activity.^76,77,78,79^ Colistin combined with antimicrobial peptides or Ethylenediaminetetra acetic acid (EDTA) has also shown effectiveness against colistin-resistant and MDR bacterial infections.^80,81^ Novel compounds currently under investigation include Fluopsin C, a secondary metabolite derived from *Streptomyces* and *Pseudomonas* species, which has shown broad-spectrum activity against both Gram-positive and Gram-negative drug-resistant bacteria.^82,83^ Similarly, Terrain, a metabolite extracted from *Aspergillus terreus*, has demonstrated potent antibacterial activity.^84^ In drug-repurposing approaches, niclosamide combined with colistin has exhibited synergistic antimicrobial effects against colistin-resistant *A. baumannii* and *K. pneumoniae*.^85^ Emerging genetic and molecular strategies, including CRISPR/Cas9-based methods, phage genome engineering,^86^ and quorum-quenching techniques,^73^ hold promise for overcoming colistin resistance and multidrug-resistant infections.

### Clinical outcomes in colistin resistance and co-resistance

Colistin resistance poses a major global health challenge, contributing to treatment failures, delays in effective therapy, prolonged hospital stays, and elevated mortality rates. *A. baumannii, K. pneumoniae*, and *E. coli* are the primary contributors to MDR infections associated with colistin resistance. Several studies have reported that pathogens demonstrated either colistin sensitivity, intermediate susceptibility or resistance against colistin along with carbapenem resistance, contributing multidrug resistance, or extensive drug resistance. These infections result in diverse clinical outcomes, including recovery, mortality, ICU admission, and extended hospitalisation, as summarised in Table 2.^87,88,89,90,91,92,93,94,95,96^

**TABLE 2:** Summary of global clinical studies on colistin resistance: Bacterial profiles, resistance rates, and clinical outcomes.

Study	Microorganisms	Resistance %	Clinical outcomes	Findings of the study
1) A prospective cross-sectional study (Tertiary hospital, Gauteng, South Africa)^87^	*A. baumannii*.	a] Col R (9.4%) b] XDR	• ICU admission (37.5%) • Mortality (47.9% at initial assessment, increasing to 52.2% at 30-day follow-up)	• All colistin-resistant *A. baumannii* isolates showed carbapenem resistance with XDR phenotype. • Mortality was higher in paediatric than in adult patients.
2) Multicentre study (Taiwan)^88^	*Acinetobacter spp*.	a] Col R (14.8%) b] Col S (85.3%)	• 14-day mortality (reported for both Col R and Col S groups)	• Higher pathogenicity and mortality rates in *A. baumannii* compared with other *Acinetobacter* spp.
3) Retrospective study (Saudi Arabia)^89^	*Enterobacteriaceae* (*n* = 227): *K. pneumoniae* (33%), *E.coli* (50%) and *Enterobacter* species (16%)	a] MDR (57.3%) b] XDR (3.52%)	• Recovery (for MDR; 40%, for XDR; 50%) • Prolonged hospital stay (for MDR; 28 days, for XDR; 20.3 days) • Mortality (for MDR; 83%, for XDR; 79%) • ICU admission [*ventilator*] (61.6% in both, MDR and XDR cases)	• High prevalence of resistant *Enterobacteriaceae* infections (*E. coli* most prevalent pathogen), associated with overall high mortality rate (84%).
4) Prospective study (Odisha, India)^90^	Gram-negative (*n* = 357) *K. pneumoniae, E. coli, P. aeruginosa, A. baumannii*	a] MDR (100%) b] Col R (19.6%)	• Recovery (74.2%) • Mortality (37.9%)	• High colistin resistance development in *K. pneumoniae* and *E. coli* among all, in samples isolated from respiratory infections and neurology ICU patients.
5) Retrospective study (Bursa, Türkiye)^91^	*Klebsiella pneumoniae*	a] CS b] CR-Col S (95%) c] CR- Col R (93%)	• For CS, Death (in 14-days and 30-days; 1.3%) • For CR-Col S, Death (in 30-days; 38.4%) • CR-Col R, Death (in 14-days and 30-days; 40.7%)	• Higher 30-days mortality in CR-Col S and CR- Col R related infections.
6) Observational study (tertiary care hospital in South India)^92^	*K. pneumoniae, A. baumannii, P. aeruginosa*	MDR + Col R	• Recovery (40%) • Mortality (60%)	• *K. pneumoniae* constituted 50%, *A. baumannii* 30%, *P. aeruginosa* 20%, with emerging colistin resistance in 16 months.
7) Retrospective study (AIDA trial in hospitals from Israel, Greece, and Italy)^93^	*Acinetobacter baumannii*	Col R – CR	• 14-day clinical failure (defined as lack of microbiological or clinical improvement) • 14-day all-cause mortality	• Development of colistin resistance during treatment of carbapenem-resistant *A. baumannii* infections was associated with higher treatment failure rates and mortality.
8) Retrospective study (tertiary care hospital in India)^94^	*Klebsiella pneumoniae*	Col R – CR	• Treatment failure • Mortality (69.3%) • ICU stay	• In hospital mortality rate was high in dual colistin and carbapenem-resistant bloodstream infections, with less survival rate in carbapenem-combination therapy.
9) Retrospective study (Pune. Maharashtra, India)^95^	*K. pneumoniae* (80%), *A. baumannii* (13.3%), *P. aeruginosa* (6.7%), and *Enterobacter cloacae* (3.3%)	Col R	• Recovery (66.7%) • Treatment failure • Mortality (33.3%)	• Colistin resistance associated mortality owing to treatment failure, in elderly patients with comorbidities and invasive procedures. • *K. pneumoniae* – predominant pathogen.
10) Retrospective study (tertiary care hospital, Chandigarh, India)^96^	*Klebsiella pneumoniae*	a] Col I (45.6 %) b] Col R (54.4 %)	In both cases: • Longer hospital stay • Mortality	• Prior hospitalisation might be contributing in emergence of colistin-resistant *K. pneumoniae* related infections even without prior use of colistin treatment and less survival rates in such patients.

Note: Please see the full reference list of Muzaheed. The colistin resistance pandemic: A dual threat from plasmid-encoded mobilised colistin resistance genes and chromosomal mutations. Afr J Lab Med. 2026;15(1), a3122. https://doi.org/10.4102/ajlm.v15i1.3122.

Col R, colistin resistant; Col S, colistin sensitive; Col I, colistin intermediate, CR, carbapenem resistant, CS, carbapenem sensitive, MDR, multidrug resistant; XDR, extensively drug resistant, ICU, intensive care unit.

## Diagnostic methods to identify colistin resistance

To detect and identify colistin-resistant bacteria rapidly from clinical, community, and environmental sources, various phenotypic and molecular methods are employed, each with its own advantages and limitations (Table 3).

**TABLE 3:** Comparative overview of phenotypic and genotypic methods for detecting colistin resistance.

Method	Principle	Turn-around time	Sensitivity / specificity (%)	Advantages	Limitations	Suitability for resource-limited settings
Broth microdilution (BMD)^118^	Reference method for MIC determination	16–24 h	100/100	CLSI/ EUCAST gold standard	Time-consuming, labour-intensive	Moderate: requires CAMHB, 96-well plates and trained personnel; reagent supply chains may be unreliable
Rapid Polymyxin NP Test^119^	Detects glucose metabolism inhibition by colistin	2–4 h	100 /95.9	Simple; inexpensive; rapid screening	May give false negatives for heteroresistant strains	High: low cost, minimal equipment; suitable for first-line screening
Lateral Flow *mcr-1* Test^107^	Immunochromatographic detection of *mcr-1* protein	< 1 h	100/98	User-friendly; suitable for low-resource settings	Detects only *mcr-1* variant	High: no equipment needed; suitable for point-of-care and field use
PCR for *mcr* genes^120,121^	Amplification of *mcr* genes	3–4 h	100/100	High accuracy; identifies gene variants	Requires technical expertise and equipment	Low to moderate: needs thermocycler, trained staff, and reagent cold chain
Whole-genome sequencing^122,123^	Detects all known resistance genes	1–2 days	100/100	Comprehensive; tracks transmission and molecular evolution	Costly; needs expert personnel; limited in heteroresistance detection	Low: high capital and running costs; requires bioinformatics capacity; best suited to reference laboratories

Note: Please see the full reference list of Muzaheed. The colistin resistance pandemic: A dual threat from plasmid-encoded mobilised colistin resistance genes and chromosomal mutations. Afr J Lab Med. 2026;15(1), a3122. https://doi.org/10.4102/ajlm.v15i1.3122.

CAMHB, cation-adjusted Mueller-Hinton broth; *mcr*, mobilised colistin resistance; PCR, polymerase chain reaction; MIC, minimum inhibitory concentration; CLSI, Clinical and Laboratory Standards Institute; EUCAST, European Committee on Antimicrobial Susceptibility Testing.

### Phenotypic detection

Several phenotypic approaches are available for antimicrobial susceptibility testing, broadly categorised as dilution-based, diffusion-based, gradient-based, and automated methods.^97^ For colistin specifically, CLSI (M100, 2025)^98^ and EUCAST (2024)^99^ endorse only the broth microdilution (BMD) method as the reference standard for susceptibility testing in Enterobacterales and other Gram-negative bacilli. Diffusion-based approaches, including disk diffusion and gradient strip tests (E-tests), are not recommended for colistin because of poor agar diffusion of the large polymyxin molecule and consequent poor reproducibility. Laboratories should therefore rely on BMD for routine colistin testing and confirm borderline results with complementary methods to ensure accuracy in clinical reporting.^100^

#### Screening with selective media

Selective media such as SuperPolymyxin^TM^, Lucie-Bardet-Jean-Marc-Rolain (LBJMR), and CHROMagar COL-APSE can be used to detect colistin-resistant *Enterobacteriaceae*. These chromogenic media inhibit the growth of fungi and Gram-positive bacteria, enabling efficient detection of resistant strains based on distinctive colony coloration. Although these methods offer high sensitivity and specificity, they are time-consuming, typically requiring 18–48 h for completion.^101,102,103^

#### Diffusion methods: Disc diffusion and Gradient methods

Disk diffusion (DD) and gradient strip tests (E-tests) are among the most widely used phenotypic antibiotic susceptibility testing (AST) approaches in clinical microbiology.^97^ In the DD method, an antibiotic-impregnated disk is placed on an inoculated Mueller–Hinton agar (MHA) plate, and the diameter of the resulting inhibition zone is measured after overnight incubation. Gradient strip tests operate on a similar diffusion principle but employ a continuous antibiotic concentration gradient along a plastic strip, with the Minimum Inhibitory Concentration (MIC) read at the intersection of the inhibition ellipse and the printed scale.^97,98^ For colistin, however, neither method is considered reliable. DD yields only categorical (qualitative) results, and both methods are compromised by the poor diffusion of the large, positively charged polymyxin molecule through agar, which is further influenced by divalent cation content, pH, and temperature.^98,104,105^ Consequently, CLSI (Clinical & Laboratory Standards Institute) and EUCAST (European Committee on Antimicrobial Susceptibility Testing) advise against their use for colistin susceptibility determination.

#### Broth microdilution method

The BMD method is the sole reference standard endorsed by both CLSI and EUCAST for colistin susceptibility testing, performed according to ISO 20776-1.^97,98,99^ Colistin-specific breakpoints have been established for key Gram-negative species. In brief, doubling dilutions of colistin sulfate are prepared in cation-adjusted Mueller–Hinton broth (CAMHB) across a 96-well microtitre plate. After inoculation with a standardised bacterial suspension and 16–18 h of incubation, the MIC is defined as the lowest colistin concentration at which no visible growth is observed.^105^ The method is accurate and well-standardised, but its manual workflow and overnight turnaround time of 16–24 h limit throughput, particularly in laboratories with high sample volumes or constrained staffing.

#### Automated system

Automated systems based on the BMD principle, such as VITEK-2, Sensititre, and BD Phoenix, can determine MIC values rapidly and facilitate quick phenotypic validation of colistin resistance.^106^

#### Lateral flow MCR-1 test

The lateral flow immunoassay allows rapid (< 15 min) detection of the MCR-1 enzyme using streptavidin-labelled monoclonal antibodies specific to MCR-1. However, this test detects only the *mcr-1 gene* variant product but not the protein products of other *mcr* genes.^107^

### Molecular detection

Molecular and genomic techniques enable direct detection of genes responsible for colistin resistance. Commonly used methods include polymerase chain reaction (PCR), whole-genome sequencing (WGS), and DNA microarray analysis.^108^

#### Polymerase chain reaction

PCR is commonly used to detect *mcr* gene variants that confer colistin resistance in bacteria. Real-time PCR facilitates the detection of *mcr* genes directly from clinical samples. This method also helps to elucidate the underlying mechanisms of colistin resistance in different bacteria and supports the surveillance of resistant isolates.^109,110^ Multiplex PCR (M-PCR) enables simultaneous detection of multiple *mcr* gene variants (*mcr-1* to *mcr-9*).^111^ A quadruplex PCR format has demonstrated the simultaneous detection of *mcr-1* through *mcr-4* in clinical samples within 4 h, supporting its use in time-sensitive surveillance contexts.^111^

#### Whole genome sequencing and sequencing platforms

Whole genome sequencing (WGS) examines the entire bacterial genome to identify both known and newly emerging resistance genes. This technique provides insights into the evolutionary pathways of resistance in bacterial strains and the distribution of known resistance genes.^112^ However, WGS is costly and has limitations in detecting heteroresistant isolates.^113^ In practice, WGS has been used to trace clonal transmission of *mcr*-carrying isolates across poultry supply chains and hospital wards, providing resolution that targeted PCR methods cannot achieve.^112,113^

Several sequencing platforms can generate WGS data for resistance surveillance. Short-read platforms such as Illumina provide high accuracy at relatively lower cost but require longer processing times and centralised infrastructure. Nanopore sequencing generates long reads in real time, which improves detection of mobile genetic elements and structural variants and allows sequencing outside conventional laboratory settings. While nanopore is not a distinct diagnostic category, its portability and decreasing cost make it worth considering for field-based surveillance in settings where laboratory access is limited.^114^

#### Deoxyribonucleic acid microarray

DNA microarray technology can identify multiple resistance genes within a short period using complementary DNA (cDNA) probes specific to resistance determinants, offering greater flexibility in detecting various mechanisms. However, this technique is expensive and requires specialised expertise.^115^

Although WGS provides comprehensive insight into the genetic basis of colistin resistance, it may fail to detect heteroresistant subpopulations below its detection threshold.^79^ A practical diagnostic workflow begins with screening using selective media or a rapid phenotypic assay, followed by confirmation with the BMD method to determine the MIC. Subsequently, PCR or WGS can be employed to identify resistance mechanisms and to monitor *mcr* gene variants for epidemiological surveillance. Integrating phenotypic and molecular data in this sequential manner enhances diagnostic accuracy and strengthens epidemiological surveillance.^105,108^

### New emerging tools

Recent advances have introduced emerging tools such as CRISPR-based assays, nanopore sequencing, and rapid phenotypic antibiotic susceptibility testing (AST) methods.

#### CRISPR-based assays

This approach employs the RPA-CRISPR/Cas12a assay for *mcr-1* gene detection, demonstrating high sensitivity and specificity. This rapid detection method enables timely identification of resistance genes in clinical settings and aids in monitoring antimicrobial resistance in veterinary and food samples, thereby supporting public health surveillance.^116^

#### Rapid antibiotic susceptibility testing detection

The Resazurin Rapid Polymyxin NP (R-RPNP) test is used to determine colistin resistance in Enterobacterales. In this assay, varying concentrations of colistin are combined with cation-adjusted Mueller–Hinton broth (CAMHB) and the Resazurin reagent to determine the MIC, based on a characteristic colour shift from blue to purple to pink.^117^ Table 3 summarises phenotypic and genotypic methods for colistin resistance detection, highlighting differences in diagnostic performance and operational feasibility.^107,118,119,120,121,122,123^

### Monitoring limitations and surveillance challenges

Despite advances in diagnostic technologies, global surveillance of colistin resistance remains inconsistent. In low- and middle-income countries, inadequate laboratory infrastructure, limited resources, insufficient funding, and a lack of governance and informatics-based systems contribute to under-detection and inconsistent reporting of resistance data. Harmonising data collection, improving comparability, and supporting timely public health interventions require increased participation of international health organisations, such as the WHO Global Antimicrobial Resistance and Use Surveillance System (GLASS), and the establishment of regional genomic monitoring networks. Effective control of AMR is not possible without addressing knowledge gaps related to both treatment practices and surveillance systems. Limitations in monitoring colistin resistance, both locally and globally, arise from multiple factors. These factors include gaps in treatment knowledge, inadequate transmission surveillance, and insufficient preventive strategies.^124^

#### Treatment-based limitations

Insufficient knowledge of best management practices (BMPs) for colistin use in clinical and veterinary settings creates information gaps and contributes to the emergence of colistin resistance in both clinical and environmental contexts. Limited understanding of the socioeconomic and behavioural factors driving antimicrobial use and misuse further hampers the identification of causes and the development of strategies to mitigate colistin resistance.^125^

#### Surveillance-based limitations

Inadequate data on the transmission of plasmid- and chromosome-mediated resistance genes, and the evolution of resistance mechanisms in bacterial strains from clinical, community, and environmental sources, create knowledge gaps regarding prevalence and global spread. The lack of rapid and effective diagnostic methods, combined with limited trained personnel, insufficient funding, and inadequate laboratory infrastructure, results in incomplete data on colistin resistance in humans, animals, and the environment. Identifying these factors is essential for implementing effective surveillance strategies to address these gaps.^125,126^

### Necessity of the One Health approach

To preserve the efficacy of colistin against infections caused by carbapenem- and β-lactam-resistant bacteria, its use as a prophylactic agent and growth promoter in livestock has been restricted or banned in many countries.^127^ In addition to the rapid spread of plasmid-mediated colistin resistance, chromosomal mechanisms also contribute to the stable transmission of resistance-conferring mutations.^8^ The presence of colistin resistance in agricultural, veterinary, and environmental sources presents significant challenges to both clinical and non-clinical settings. Therefore, a One Health approach should be implemented at local, national, and global levels to address this issue. Given the link between antibiotic use and the emergence of colistin resistance, it is essential to regulate its use in both clinical and non-clinical settings.^128^ Controlling its spread through food, animals, humans, and the environment requires robust surveillance and effective preventive strategies.

In the African context, practical implementation of One Health surveillance could draw on structures that already exist. The Africa CDC AMR Surveillance Network provides a framework for coordinating human health data across the continent, while the animal health programmes and national veterinary services of AU-IBAR offer entry points for agricultural and livestock monitoring.^129,130,131^

Regional economic communities including ECOWAS (Economic Community of West African States), EAC (East African Community), SADC (Southern African Development Community), and IGAD (Intergovernmental Authority on Development) could play a role in harmonising policies across national borders. Livestock, and particularly small ruminants and poultry, are central to the livelihoods of large populations across Africa, and their role as reservoirs of colistin-resistant organisms owing to informal antibiotic use means these animals cannot be excluded from any meaningful surveillance framework.^132,133^

Rapid detection and the implementation of effective treatment regimens, guided by clinical assessment of colistin resistance, are essential for diagnostic and therapeutic strategies. Effective surveillance must integrate clinical, veterinary, and environmental data within a One Health framework to monitor the global emergence of resistance. In addition, global efforts must include social awareness initiatives and sustainable financial support to implement this approach effectively.^8,127^

### Challenges for colistin susceptibility testing in African laboratories

Implementing the recommended BMD method for colistin susceptibility testing presents real practical difficulties for many African laboratories. The method requires cation-adjusted Mueller–Hinton broth, sterile 96-well microtitre plates, and accurately calibrated bacterial inocula, all of which may be costly, intermittently available, or simply absent in public-sector facilities. In this context, many clinical laboratories continue to rely on disk diffusion or E-test methods, largely because these methods are cheaper and more accessible, despite the fact that neither is recommended for colistin by CLSI or EUCAST.^99,100^

Beyond the gold standard method, access to molecular platforms for *mcr* gene detection is limited across much of the continent. Trained microbiologists and laboratory scientists are in short supply, external quality assurance programmes for colistin testing are not widely available, and supply chains for reagents are frequently disrupted. The lateral flow *mcr-1* immunoassay offers a lower resource option for detecting the most prevalent variant, although it does not detect other *mcr* genes.^107^ Strengthening African laboratory capacity will require targeted investment in training, affordable equipment, and integration into regional reference networks, as well as support for participation in WHO GLASS (Global Antimicrobial Resistance and Use Surveillance System) reporting.

## Conclusion and future directions

The rapid plasmid-mediated horizontal transfer and recurrence of chromosome-mediated colistin-resistant infections pose a serious global threat. The continuous evolution of plasmid-mediated and chromosomal resistance contributes to treatment failures, prolonged hospital stays, high mortality and increased healthcare costs. Addressing this challenge requires sustained research and well-structured policy planning focused on key priorities. Specifically, it is necessary to restrict colistin use in agriculture and veterinary practice to reduce selective pressure, enhance rapid and accurate genomic and epidemiological surveillance for *mcr* genes and chromosomal mutations, and continue research into the molecular and evolutionary mechanisms underlying emerging resistance. To prevent transmission between animals, humans, and the environment, there is an urgent need to develop rapid, cost-effective, and accurate phenotypic and molecular detection methods, coupled with comprehensive surveillance strategies covering both clinical and non-clinical settings. Policies promoting antimicrobial stewardship to ensure judicious colistin use, along with effective surveillance strategies, are essential to curb the spread of colistin resistance. Future research should focus on affordable, field-deployable molecular assays and global genomic surveillance to track resistance evolution within a One Health framework, enabling effective management at both social and economic levels.

For African countries, a number of priority actions stand out. First, laboratory capacity for BMD testing needs to be built through training programmes, equipment provision, and the development of regional reference laboratories. Second, national and regional surveillance systems should be established for *mcr* gene variants covering clinical, veterinary, and environmental isolates. Third, human and animal health data should be integrated under One Health frameworks that draw on the structures of Africa CDC and AU-IBAR. Fourth, African laboratories and health ministries should be supported in participating in WHO GLASS (Global Antimicrobial Resistance and Use Surveillance System). Fifth, the development of diagnostic tools that are affordable and deployable outside centralised laboratory settings deserves targeted research investment. These are not aspirational goals in the distant future; they are achievable with the right partnerships and commitment.

Implementing robust strategies will support the development of a comprehensive framework for surveillance, management, treatment, and prevention of colistin-resistant infections, addressing treatment failures and reducing the threat to human, animal, and environmental health, ultimately improving public health outcomes.
